# Impact of undiagnosed obstructive sleep apnea on atrial fibrillation recurrence following catheter ablation (OSA-AF study)^☆^

**DOI:** 10.1016/j.ijcha.2022.101014

**Published:** 2022-03-24

**Authors:** John de Heide, Danielle B.M. Kock-Cordeiro, Rohit E. Bhagwandien, Mark G. Hoogendijk, Koen C. van der Meer, Sip A. Wijchers, Tamas Szili-Torok, Felix Zijlstra, Mattie J. Lenzen, Sing-Chien Yap

**Affiliations:** a Department of Cardiology, Erasmus MC, University Medical Center Rotterdam, Rotterdam, the Netherlands; b Department of Intensive Care/Home Mechanical Ventilation and Pulmonology, Erasmus MC, University Medical Center Rotterdam, Rotterdam, the Netherlands

**Keywords:** Obstructive sleep apnea, Atrial fibrillation, Catheter ablation

## Abstract

**Background:**

Sleep-disordered breathing (SDB) may hamper the outcome of catheter ablation of atrial fibrillation (AF). However, SDB is underdiagnosed in clinical practice and the relevancy of undiagnosed SDB on the outcome of catheter ablation is unclear.

**Objective:**

To evaluate if undiagnosed SDB has an impact on AF recurrence after catheter ablation.

**Methods:**

In this single-center cohort study we enrolled patients who had a catheter ablation of AF 12 to 18 months prior to enrolment. Patients with diagnosed SDB at the time of catheter ablation were excluded. Enrolled patients underwent screening using WatchPAT (WP). SDB was defined as an apnea-hypopnea index (AHI) ≥ 15.

**Results:**

A total of 164 patients were screened for eligibility. After exclusion of patients with previously diagnosed SDB (n = 30), 104 of 134 eligible patients were enrolled and underwent SDB screening. The median AHI was 11.5 (interquartile range 6.8–21.9) and 39 patients (38%) had SDB which was undiagnosed during the first year after ablation. AF recurrence in the first year after catheter ablation occurred in 40 patients (38%). The risk of AF recurrence was higher in the group with undiagnosed SDB in comparison to those without SDB (51% versus 31%, P = 0.04). Interestingly, the prevalence of AF recurrence was similar between patients with previously diagnosed and undiagnosed SDB (51% versus 50%, P = 0.92).

**Conclusion:**

A significant proportion of patients undergoing catheter ablation of AF have undiagnosed SDB which is associated with a twofold higher risk of AF recurrence. SDB screening may improve patient counselling regarding the efficacy of catheter ablation.

## 1 Introduction

There is an association between sleep-disordered breathing (SDB) and atrial fibrillation (AF). The putative mechanisms for AF vulnerability in SDB patients seem to be a combination of left atrial (LA) dilatation, altered autonomic nerve activity, neurohumoral activation and electrical atrial remodeling (e.g., atrial conduction slowing, reduction of atrial effective refractory period) [1]. Observational studies and *meta*-analyses have shown a negative impact of SDB on the efficacy of catheter ablation of AF, with a 25% increased risk of AF recurrence [2], [3], [4], [5], [6]. This lower efficacy may be partly explained by an increased incidence of non-pulmonary vein triggers [7]. Treatment of SDB with continuous positive airway pressure (CPAP) improves arrhythmia-free survival after catheter ablation in observational studies, with a 42% risk reduction of AF recurrence [8], [9], [10]. The 2020 European Society of Cardiology (ESC) guidelines recommend that SDB treatment should be optimized to improve AF treatment results [11], [12].

The clinical challenge, however, is that many patients with SDB have limited symptoms such as daytime sleepiness or feelings of fatigue which results in underdiagnosis of SDB [13], [14], [15]. At present, the role of opportunistic screening for SDB before catheter ablation of AF is unclear. The aim of the current study is to evaluate if undiagnosed, thus untreated, SDB was associated with AF recurrence within the first year after initial catheter ablation of AF. To prevent bias due to SDB treatment, we evaluated the presence or absence of SDB, as measured with a dedicated sleep apnea testing device, at least one year after the initial catheter ablation. In addition, we also tested the utility of commonly used SDB screening questionnaires such as STOP-BANG and the Epworth Sleepiness Scale (ESS) to predict SDB in this specific patient population.

## 2 Methods

### 2.1 Study population

The *Effect of undiagnosed Obstructive Sleep Apnea in patients undergoing Atrial Fibrillation catheter ablation* (OSA-AF) study was a cross-sectional single-center cohort study. We included consecutive patients after a first catheter ablation of AF in the Erasmus MC, University Medical Center Rotterdam, the Netherlands from December 2018 to February 2020. Eligible patients were adults who had a first catheter ablation of AF 12 to 18 months prior to screening for SDB. Thus, SDB status was determined at least 12 months after catheter ablation. We used the assumption that SDB status determined between 12 and 18 months after catheter ablation would reflect the SDB status at the time of catheter ablation. Using this unique study design we prevented treatment bias, which can occur when SDB status was determined at the time of catheter ablation. Consecutive patients were approached during their regular follow-up at the outpatient clinic. We excluded patients who had previously diagnosed SDB at the time of the catheter ablation.

### 2.2 Assessment of AF recurrence

After a catheter ablation of AF, patients had a routine follow-up at 3, 6 and 12 months after their ablation. Routinely, a 24-hour Holter monitoring was performed at 3 and 6 months, and a 7-day Holter monitoring was performed at 12 months after ablation. Additional Holter monitoring was performed when necessary. AF recurrence was defined as documented AF > 30 s at Holter monitoring or documented AF on a standard 12-lead ECG, after a blanking period of 3 months, irrespective of the use of antiarrhythmic drugs.

### 2.3 Screening for SDB

**Fig. 1:**
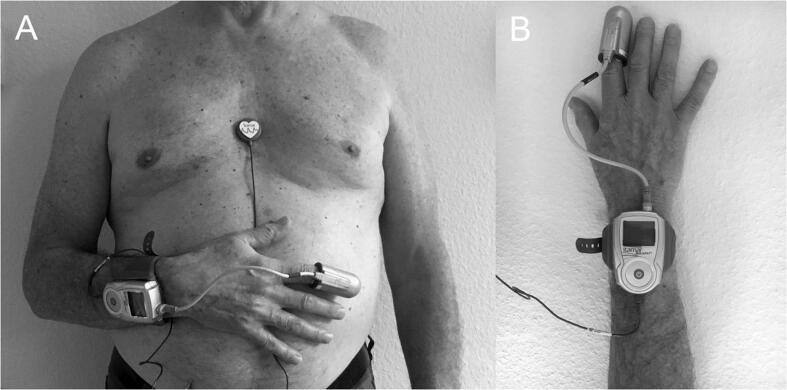
WatchPAT-200U system: position of snoring and body position sensor on sternum (A) and position of WatchPAT wrist unit and finger probe (B).

Screening for SDB was performed using the WatchPAT-200U (WP) (Itamar Medical, Caesarea, Israel). The WP system is a home sleep apnea testing (HSAT) device which has been shown to be accurate for diagnosing SDB, also in patients with AF [16]. It consists of a wrist-worn device with a finger probe that obtains peripheral arterial tonometry (PAT) signals and oxygen saturation levels, a snoring and body position sensor that is positioned under the sternal notch and accelerometer that is embedded in the wrist unit (Fig. 1). The WP finger probe measures changes in the vascular tone at the fingertip which is a measure of sympathetic nervous system activity. Respiratory events are typically terminated by sympathetic activation and this is reflected by transient vasoconstriction events and increased pulse rate [17]. The WP algorithm detects respiratory (apnea/hypopnea) events, sleep/wake status, and determines sleep stages. Patients were instructed in the use of the device and used the WP device overnight. If patients had questions on the use of the WP system, they could easily contact us by phone or email. For this study, SDB was defined as an apnea-hypopnea index (AHI) ≥ 15. If the proportion of central apneas over the total number of apneas was ≥ 50%, these patients were considered to have predominant central sleep apnea; otherwise, they had predominant obstructive sleep apnea.

### 2.4 SDB screening questionnaires

Enrolled patients were requested to complete two questionnaires: the STOP-BANG and ESS questionnaire. The STOP-BANG questionnaire is specifically developed as a screening tool for SDB [18]. It consists of 8 dichotomous items related to the clinical features of SDB, with a total score ranging from 0 to 8. Intermediate risk is defined as 3–4 points, and high risk is defined as ≥ 5 points or as 2 points in the first 4 questions in combination with male sex, obesity (BMI > 35 kg/m^2^) or wide neck circumference (>40 cm females, >42 cm males). An intermediate or high-risk STOP-BANG score was considered abnormal in this study.

The ESS is a validated questionnaire to screen for excessive daytime sleepiness, which is an important symptom to refer patients for SDB screening [19]. It consists of 8 questions related to falling asleep in several common situations, each scored with a degree of severity ranging from 0 to 3. Scores range from 0 (least sleepy) to 24 (sleepiest). Excessive daytime sleepiness was defined in this study as an ESS ≥ 11.

### 2.5 Statistical analysis

Continuous parameters are presented as mean ± standard deviation (SD) or as median and interquartile range (IQR), as appropriate. Categorical data are presented as frequencies and percentages. Comparisons between groups were performed with an independent Student *t*-test, Mann-Whitney *U* test, chi-square test, or Fisher exact test, where appropriate. In case of comparing>2 groups, one-way ANOVA or Kruskal-Wallis test was used to compare continuous variables, where appropriate. Outcomes of logistic regression analysis are presented as odds ratios (OR) and 95% confidence intervals (CI). A P-value < 0.05 was considered statistically significant. Statistical analyses were performed using SPSS software (SPSS, version 25; IBM, Chicago, Illinois).

### 2.6 Ethics

The Medical Ethics Committee reviewed the study (MEC-2018–1503), and this single-center cohort study was not subjected to the Dutch Medical Research Involving Human Subjects Act. All participants undergoing SDB screening provided written informed consent. There was a waiver for the use of retrospective data. The study was carried out according to the ethical principles for medical research involving human subjects established by Declaration of Helsinki, protecting the privacy of all the participants and the confidentiality of their personal information.

## 3 Results

### 3.1 Patient population

**Fig. 2:**
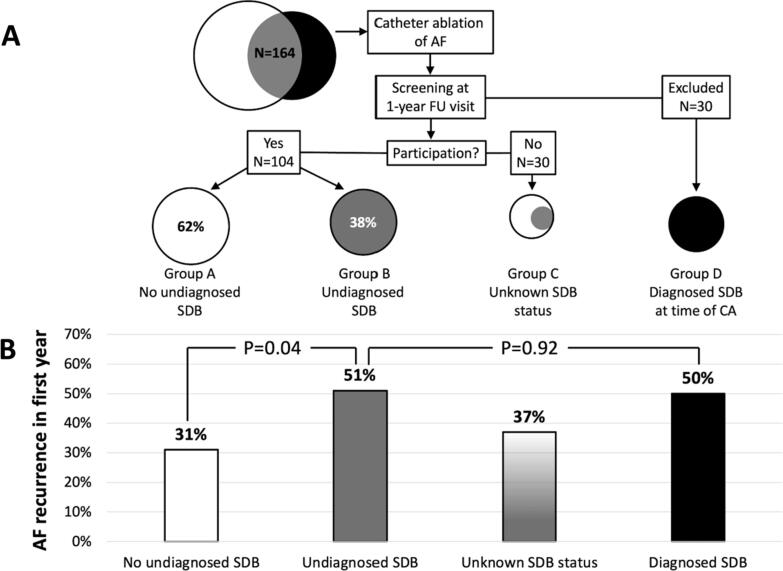
Study flow chart (A) and AF recurrence rate in the first year after catheter ablation per group (B). CA = catheter ablation; FU = follow-up; SDB = sleep-disordered breathing.

A total of 164 consecutive patients were scheduled for a 1-year follow-up visit at the outpatient clinic after their first catheter ablation of AF (Supplemental Table 1). After exclusion of 30 patients with diagnosed SDB at the time of catheter ablation, 104 of 134 patients (participation rate 78%) were enrolled and comprised the final study population (Fig. 2A). Patient characteristics of the study population are presented in Table 1. In the final study population, there were 40 patients (38%) with AF recurrence in the first year after catheter ablation. All patients could successfully use the WatchPAT, there were no dropouts.

**Table 1:** Patient characteristics.

**Characteristic**	**Total** **N = 104**	**Group A** **No SDB** **N = 65**	**Group B** **Undiagnosed SDB** **N = 39**	**P-value**
**Demographic data**				
Age, years	59 ± 10	57 ± 9	62 ± 9	0.01
Female sex	34 (33)	15 (23)	19 (49)	0.01
**Type of AF at index procedure**				
Paroxysmal AF	77 (74)	52 (80)	25 (64)	0.07
Nonparoxysmal AF	27 (26)	13 (20)	14 (36)	0.07
**LA size**				
LAVI, ml/m^2^	38 ± 14	36 ± 14	41 ± 14	0.07
**Scores**				
CHA_2_DS_2_-VASc	1.4 ± 1.3	1.0 ± 1.0	2.1 ± 1.3	<0.001
CHA_2_DS_2_-VASc ≥ 2	44 (42)	18 (28)	26 (67)	<0.001
**Modifiable risk factors**				
Obesity, BMI ≥ 30 kg/m^2^	20 (19)	9 (14)	11 (28)	0.07
BMI	26.5 (24.4–29.2)	25.7 (24.2–28.8)	27.8 (26.4–31.1)	0.02
Diabetes	7 (7)	1 (2)	6 (15)	0.01
Hyperlipidaemia	12 (12)	7 (11)	5 (13)	0.76
Hypertension	42 (40)	15 (23)	27 (69)	<0.001
Smoking	6 (6)	3 (5)	3 (8)	0.67
Alcohol use*	9 (9)	3 (5)	6 (15)	0.08
**Type of procedure**				
PVI only	96 (92)	60 (92)	36 (92)	1.00
PVI and substrate ablation	8 (8)	5 (8)	3 (8)	1.00
**Antiarrhythmic drugs**				
None	34 (33)	28 (43)	6 (15)	0.004
Flecainide	21 (20)	13 (20)	8 (21)	0.95
Betablockers	34 (33)	18 (28)	16 (41)	0.16
Sotalol	25 (24)	12 (19)	13 (33)	0.09
Amiodarone	2 (2)	1 (2)	1 (3)	0.71
Verapamil	4 (4)	2 (3)	2 (5)	0.58
Digoxin	3 (3)233	1 (2)	2 (5)	0.28

Data are presented as mean ± SD, median (IQR) or as n (%). AAD = antiarrhythmic drug; AF = atrial fibrillation; AHI = apnea-hypopnea index; BMI = body mass index; LA = left atrial; LAVI = left atrial volume index; PVI = pulmonary vein isolation; SDB = sleep-disordered breathing.

* Alcohol use was defined as > 1 standard drink per day.

### 3.2 SDB status and risk of AF recurrence

**Fig. 3:**
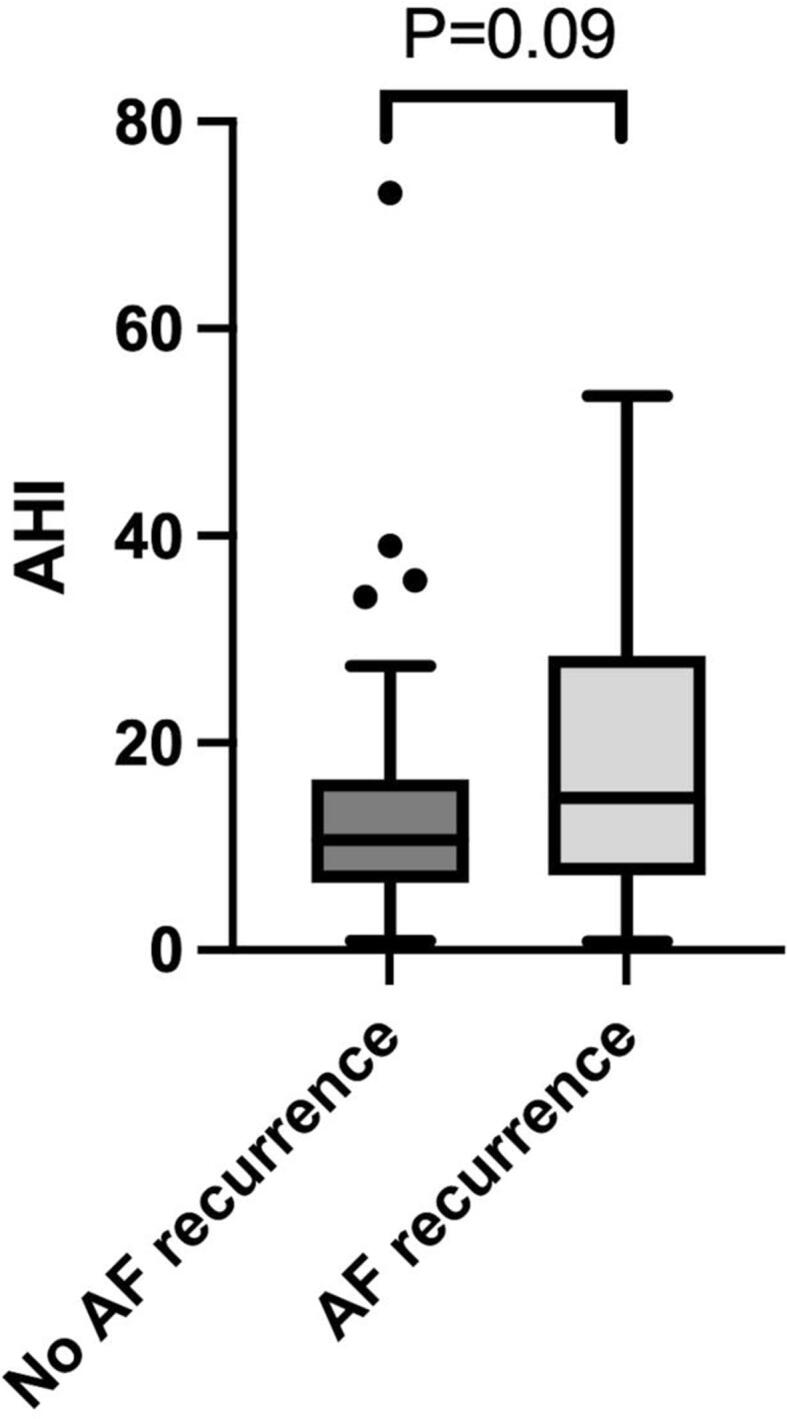
Tukey box plots demonstrating the apnea-hypopnea index (AHI) for patients with and without AF recurrence. The whiskers are defined as 1.5 * interquartile range. Outliers are denoted by the dots. AF = atrial fibrillation; AHI = apnea-hypopnea index.

The median WP-derived AHI for the total study population was 11.5 (IQR, 6.8–21.9) and 39 patients (38%) had undiagnosed SDB with an AHI ≥ 15. All patients with undiagnosed SDB (AHI ≥ 15) had predominant obstructive sleep apnea (no patient had predominant central sleep apnea), with a very low median central AHI of 1.9 (IQR, 0.6–4.4). The patient characteristics between patients with and without undiagnosed SDB is presented in Table 1. In comparison with patients with no SDB, patients with undiagnosed SDB were older, were more often female, more often had diabetes and hypertension, higher CHA_2_DS_2_-VASc score, higher body mass index and more often used antiarrhythmic drugs (Table 1). The risk of AF recurrence was higher in patients with undiagnosed SDB in comparison to patients without undiagnosed SDB (51% versus 31%, OR 2.37, 95% CI 1.04–5.38, P = 0.04) (Fig. 2B). Vice versa, patients with AF recurrence showed a trend towards a higher median AHI value, 14.7 (IQR, 7.5–28.0) versus 10.6 (IQR, 6.6–16.5), P = 0.09 (Fig. 3).

The risk of AF recurrence was similar between patients with undiagnosed and previously diagnosed SDB (51% versus 50%, OR 1.05, 95% CI 0.41–2.73, P = 0.92) (Fig. 2B). Patient characteristics between patients with undiagnosed and previously diagnosed SDB were similar, except patients with previously diagnosed SDB more often used amiodarone (Supplemental table 1).

### 3.3 Performance of SDB screening questionnaires

**Fig. 4:**
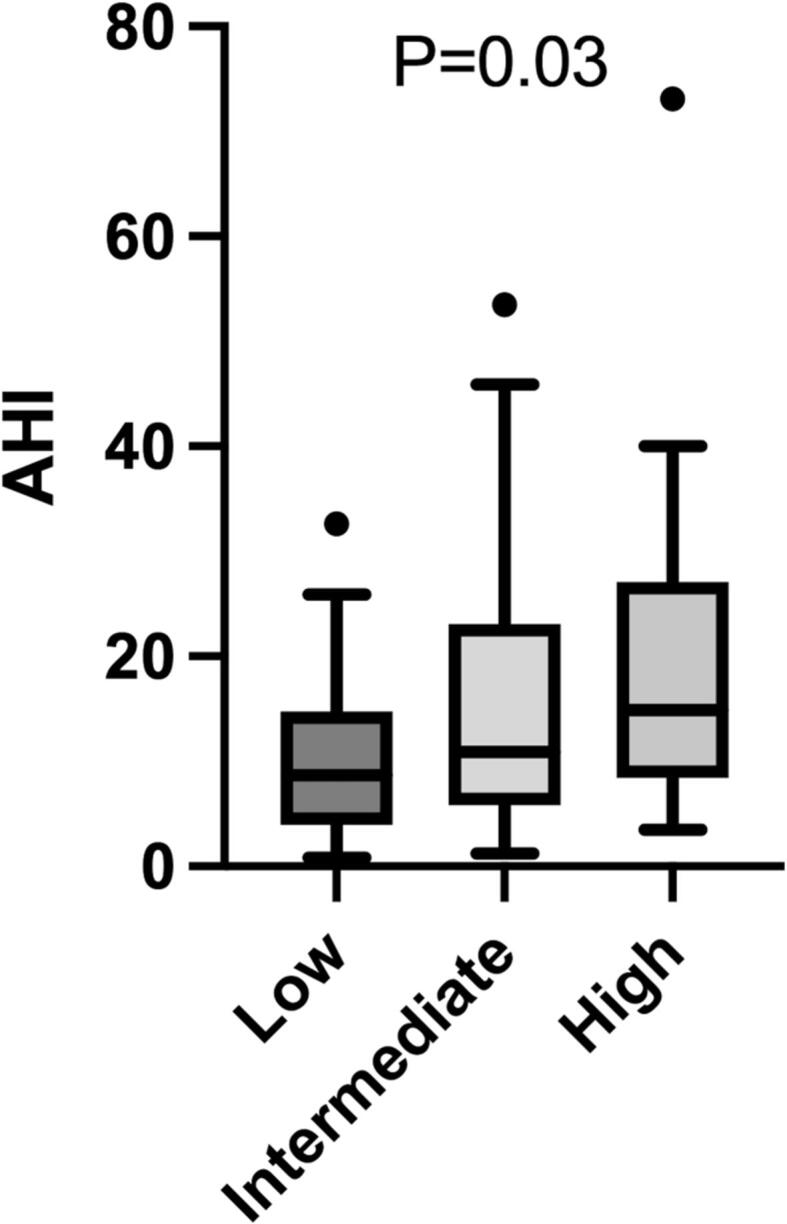
Tukey box plots demonstrating the apnea-hypopnea index (AHI) for patients with low, intermediate, and high-risk STOP-BANG score. The whiskers are defined as 1.5 * interquartile range. Outliers are denoted by the dots and triangles. AHI = apnea-hypopnea index.

In total, 95 (91%) and 102 (98%) patients completed the STOP-BANG questionnaire and the ESS questionnaire, respectively. An abnormal STOP-BANG score (intermediate or high-risk score) was present in 61 patients (64%). A higher proportion of patients with SDB had an abnormal STOP-BANG score in comparison to patients without SDB (79% versus 56%, P = 0.02). An abnormal STOP-Bang score had a sensitivity of 79% and specificity of 44% for the detection of SDB (AHI ≥ 15) with a positive predictive value (PPV) of 44% and negative predictive value (NPV) of 79%. The diagnostic accuracy of the test was 57%. The area under the receiver operating characteristic curve (AUC) was 0.62 denoting a poor diagnostic discrimination. When using only the high-risk STOP-BANG score, the sensitivity, specificity, PPV, NPV and diagnostic accuracy were 35%, 76%, 44%, 68%, and 61%, respectively. Thus, specificity improved at the expense of sensitivity. Fig. 4 provides a comparison of the AHI values for the different STOP-BANG classifications. The median AHI values were statistically different between STOP-BANG groups: patients with low, intermediate, and high-risk STOP-BANG score had a median AHI of 8.7 (IQR, 4.1–14.3), 10.9 (IQR, 6.0–22.8), and 14.9 (IQR, 9.1–24.5), respectively (P = 0.03).

The mean ESS score was 4.0 ± 3.6. Excessive daytime sleepiness (ESS ≥ 11) was present in 7 patients (7%). There was no difference in excessive daytime sleepiness between patients with and without SDB (8% versus 6%, P = 0.75). Using an ESS score ≥ 11, the sensitivity was 8% and the specificity was 94% for detecting SDB (AHI ≥ 15), with a PPV of 43% and NPV of 63%. The diagnostic accuracy of an ESS score ≥ 11 was 62%. The AUC was 0.51 denoting a poor diagnostic discrimination.

## 4 Discussion

The present study demonstrates that a large proportion of patients undergoing catheter ablation of AF have undiagnosed SDB. In this specific population, the STOP-Bang and ESS questionnaires do not accurately predict the presence of SDB. Importantly, undiagnosed SDB was associated with a two-fold higher risk of AF recurrence in the first year after catheter ablation.

SDB is considered a modifiable risk factor for AF [1]. The combination of LA remodelling and deranged neurohumoral and autonomic nervous activity seems to be responsible for the increased vulnerability for AF in SDB patients. Adequate treatment of SDB may reduce the development of AF and reduce AF burden. Randomized controlled trials have shown that aggressive treatment of modifiable risk factors for AF, including SDB, successfully reverses early onset AF [20], [21], [22]. As SDB shares the same modifiable risk factors as AF, being hypertension, smoking, diabetes, hyperlipidaemia, alcohol, obesity, and physical inactivity [20], aggressive risk factor management may have a positive influence on both entities.

In the clinical context of catheter ablation, the presence of SDB may also be important. Previous studies have shown a negative impact of SDB on the efficacy of catheter ablation of AF, with a 25% increased risk of AF recurrence [2], [3], [4], [5], [6]. Interestingly, a *meta*-analysis demonstrated that SDB diagnosed by polysomnography (PSG) was a strong predictor of AF recurrence after catheter ablation, but not when SDB was diagnosed by the Berlin questionnaire [6]. This suggests that the method of SDB screening is relevant to predict AF recurrence. Furthermore, treatment of appropriately diagnosed SDB with CPAP improves arrhythmia-free survival after catheter ablation in observational studies [8], [9], [10].

The challenge is that screening for SDB is suboptimal in clinical practice. A large majority of patients with SDB remain undiagnosed as demonstrated by our study and others [13], [14], [15]. Screening for SDB can be done with questionnaires (e.g., Berlin, STOP-BANG, ESS) but the accuracy of these questionnaires is limited, especially in patients with cardiovascular disease [13], [23], [24]. Kadhim et al. previously demonstrated that excessive daytime sleepiness (ESS ≥ 11) was present in only 22 of 149 ambulatory patients (15%) with AF and moderate-to-severe SDB (AHI ≥ 15, assessed by PSG) [13]. This low prevalence of excessive daytime sleepiness is also seen in our population and thus daytime sleepiness should not be used in clinical practice to select patients for SDB screening. Even more dedicated questionnaires such as STOP-BANG do not accurately predict SDB although patients with SDB did more often had an abnormal STOP-BANG score [24]. Further research is needed before deciding on the most optimal screening method and the role of questionnaires. Currently, it is not clear whether it is cost-effective to perform SBD testing in every patient who is eligible for catheter ablation. Maybe it is more cost-effective to perform SBD testing only in patients with an intermediate to high probability based on questionnaires.

Opportunistic screening for SDB of eligible patients for catheter ablation of AF with PSG does not seem realistic in clinical practice. In this respect, HSAT seems to be easier to implement as part of the diagnostic work-up for a catheter ablation. Respiratory indexes calculated using PAT-based HSAT devices, such as the WatchPAT, correlate positively with PSG [25]. In our study of patients undergoing catheter ablation of AF, 38% of patients had newly diagnosed SDB. This prevalence is higher than a previously reported study where the prevalence of SDB was 18% when diagnosed by PSG [26]. However, a recent study by Verhaert et al., which also used WatchPAT for SDB screening, demonstrated that 55% of patients scheduled for catheter ablation had moderate-to-severe SDB (AHI ≥ 15) [27]. Furthermore, this study demonstrated that WatchPAT allows easy implementation of sleep apnea management in an AF outpatient clinic.

Before starting opportunistic screening for SDB in patients undergoing catheter ablation of AF, we first wanted to evaluate the impact of undiagnosed SDB on the outcome of catheter ablation. Our study demonstrates that undiagnosed SDB was associated with a two-fold increased risk of AF recurrence after catheter ablation of AF. These data are important for patient counselling regarding the efficacy of catheter ablation of AF. Based on the current study, we cannot rule out that SDB is merely a risk marker than a risk factor. A risk marker can be considered a risk factor if intervention (e.g., CPAP) to modulate this factor results in parallel modulation of risk (i.e., reduction of AF recurrence). A recent randomized controlled trial by Traaen et al. demonstrated that treatment with CPAP for 5 months did not reduce AF burden in patients with paroxysmal AF and moderate to severe SDB (AHI ≥ 15) [28]. Currently, there is no randomized controlled trial which has demonstrated the effect of CPAP use on AF recurrence after catheter ablation in SDB patients. Interestingly, in our study the risk of AF recurrence in the group with undiagnosed SDB was as high as those with previously diagnosed SDB (Fig. 2B). It may be presumed that patients with previously diagnosed SDB received appropriate SDB treatment, but we have no data on the type of treatment these patients received.

### 4.1 Study limitations

We did not determine SDB at the time of the index procedure to prevent treatment bias (i.e., CPAP treatment may influence the rate of AF recurrence). However, there are inherent limitations to our study design. There is a potential influence of catheter ablation on the prevalence and severity of SDB. Naruse et al. demonstrated that successful catheter ablation of AF reduced AHI one week after ablation [29]. It was hypothesized that reduced airway congestion due to restoration of sinus rhythm would decrease AHI. In contrast, Hoyer et al. demonstrated that catheter ablation had no influence on the prevalence and severity of SDB 6 months after the procedure [30]. We determined AHI 12 to 18 months after catheter ablation, and it is unknown how good this correlates with AHI at the index procedure. An alternative would have been to screen for SDB at the time of the index procedure in a double-blind fashion (patients and physicians unaware of SDB status) for the first year after ablation. Finally, an important limitation is that we used the WatchPAT and not PSG as the gold standard to diagnose SDB. Despite the good correlation between WatchPAT and PSG, this may have influenced the results of our study.

## 5 Conclusions

Undiagnosed SDB is common in patients undergoing catheter ablation of AF and is associated with a two-fold increased risk of AF recurrence. Screening for SDB in patients eligible for catheter ablation of AF may improve patient counselling with respect to the efficacy of catheter ablation. A HSAT-device may be a useful and easy to implement tool to screen for SDB.

## Funding

None.

## Declaration of Competing Interest

The authors declare that they have no known competing financial interests or personal relationships that could have appeared to influence the work reported in this paper.
