# Supplementary material for: Impact of undiagnosed obstructive sleep apnea on atrial fibrillation recurrence following catheter ablation (OSA-AF study)
Source: Int J Cardiol Heart Vasc. 2022 Mar 24;40:101014. doi: 10.1016/j.ijcha.2022.101014 (PMC9157450; doi:10.1016/j.ijcha.2022.101014)
Supplement: Supplementary data 1 [file mmc1.docx]

**Supplemental table 1.** Patient characteristics of all screened patients.

| **Characteristic** | **Total**  **N = 164** | **Group A**  **No SDB**  **N=65** | **Group B**  **Undiagnosed SDB**  **N=39** | **Group C**  **Unknown SDB status**  **N=30** | **Group D**  **Previously diagnosed SDB**  **N=30** | **P-value** |
| --- | --- | --- | --- | --- | --- | --- |
| **Demographic data** |  |  |  |  |  |  |
| Age, years | 59 ± 9 | 57 ± 9 | 62 ± 9 | 59 ± 11 | 63 ± 7 | <0.01^a,c,f^ |
| Female sex | 51 (31) | 15 (23) | 19 (49) | 8 (27) | 9 (30) | 0.048^a^ |
| **Type of AF at index procedure** |  |  |  |  |  |  |
| Paroxysmal AF | 119 (73) | 52 (80) | 25 (64) | 23 (77) | 19 (63) | 0.19 |
| Nonparoxysmal AF | 45 (27) | 13 (20) | 14 (36) | 7 (23) | 11 (37) | 0.19 |
| **LA size** |  |  |  |  |  |  |
| LAVI, ml/m^2^ | 39 ± 15 | 35 ± 14 | 41 ± 14 | 41 ± 18 | 41 ± 16 | 0.20 |
| **Scores** |  |  |  |  |  |  |
| CHA_2_DS_2_-VASc | 1.5 ± 1.3 | 1.0 ± 1.0 | 2.1 ± 1.3 | 1.3 ± 1.3 | 1.7 ± 1.3 | <0.01^a,c,d^ |
| CHA_2_DS_2_-VASc ≥2 | 68 (42) | 18 (28) | 26 (67) | 9 (30) | 15 (50) | <0.01^a,c,d^ |
| **Modifiable risk factors** |  |  |  |  |  |  |
| Obesity, BMI ≥30 kg/m^2^ | 39 (24) | 9 (14) | 11 (28) | 11 (37) | 8 (27) | 0.08 |
| BMI | 27.2 (24.7-29.8) | 25.7  (24.2-28.8) | 27.8  (26.4-31.1) | 28.3 (25.3-31.9) | 28.2  (26.0-30.0) | 0.02^a,c^ |
| Diabetes | 14 (9) | 1 (2) | 6 (15) | 3 (10) | 4 (13) | 0.06 |
| Hyperlipidaemia | 28 (17) | 7 (11) | 5 (13) | 7 (23) | 9 (30) | 0.08 |
| Hypertension | 67 (41) | 15 (23) | 27 (69) | 9 (30) | 16 (53) | <0.01^a,c,d^ |
| Smoking | 12 (7) | 3 (5) | 3 (8) | 3 (10) | 3 (10) | 0.72 |
| Alcohol use* | 20 (12) | 3 (5) | 6 (15) | 4 (13) | 7 (23) | 0.06 |
| **Type of procedure** |  |  |  |  |  |  |
| PVI only | 144 (88) | 60 (92) | 36 (92) | 23 (77) | 25 (83) | 0.11 |
| PVI and substrate ablation | 20 (12) | 5 (8) | 3 (8) | 7 (23) | 5 (17) | 0.11 |
| **Anti-arrhythmic drug** |  |  |  |  |  |  |
| None | 38 (23) | 28 (43) | 6 (15) | 3 (10) | 1 (3) | <0.01^a,b,c^ |
| Flecainide | 35 (21) | 13 (20) | 8 (21) | 8 (27) | 6 (20) | 0.89 |
| Betablockers | 58 (36) | 18 (28) | 16 (41) | 14 (47) | 10 (33) | 0.27 |
| Sotalol | 46 (28) | 12 (19) | 13 (33) | 10 (33) | 11 (37) | 0.17 |
| Amiodarone | 12 (7) | 1 (2) | 1 (3) | 4 (13) | 6 (20) | <0.01^b,c,e^ |
| Verapamil | 8 (5) | 2 (3) | 2 (5) | - | 4 (13) | 0.09 |
| Digoxin | 4 (2) | 1 (2) | 2 (5) | 1 (3) | - | 0.52 |

Data are presented as mean ± SD or as n (%). AAD = antiarrhythmic drug; AF = atrial fibrillation; AHI = apnea-hypopnea index; BMI = body mass index; LA = left atrial; LAVI = left atrial volume index; PVI = pulmonary vein isolation; SDB = sleep-disordered breathing. *Alcohol use was defined as >1 standard drink per day.

^a^ = statistically significant difference between group A versus B, ^b^ = group A versus C, ^c^ = group A versus D, ^d^ = group B versus C, ^e^ = group B versus D, ^f^ = group C versus D
